# A Report on the Satisfactory Results with Intra-Muscular Injections of the Salicylate of Mercury in the Treatment of Syphilis

**Published:** 1907-06

**Authors:** Edgar G. Ballenger

**Affiliations:** Lecturer on Genito-Urinary Diseases, Atlanta School of Medicine, Atlanta, Ga.


### *A REPORT ON THE SATISFACTORY RESULTS WITH INTRA-MUSCULAR INJECTIONS OF THE SALICYLATE OF MERCURY IN THE TREATMENT OF SYPHILIS.*

By Edgar G. Ballenger, M. D.,
Lecturer on Genito-Urinary Diseases, Atlanta School of Medicine,
Atlanta, Ga.

For two years I have been using the intra-muscular injections of salicylate of mercury in the treatment of syphilis with such excellent results where internal medication and inunctions had failed that I desire to report the history of a few illustrative cases and describe briefly this method of treatment.

1. Mr. A. age 34, contracted syphilis about three months before applying to me for treatment and had been under the care of an eclectic physician in south Georgia, who gave him large doses of "vegetable" drugs and iodide of potash, but in spite of the use of these, he steadily grew worse and when he presented himself there were four distinct chancres, much indurated and inaccessible on account of a phimotic prepuce. They had developed successively within two weeks after the initial lesion. His skin was covered with the most extensive macaular eruption I have ever seen, from his neck to his feet being almost a solid brick dust color. The lymphatic glands were enlarged and there was a rise of temperature of several degrees every night, causing considerable insomnia. Altogether his symptoms suggested a formidable type of syphilis.

At his first visit I injected 9-10 of a grain of salicylate of mercury in the muscles of the buttock and requested that he return in 5 days. I then made arrangements to have his photograph taken on his next visit, as I thought it would be of interest on account of the severity of the skin eruption. Very much to my surprise when he reported on the fifth day after the treatment, three-fourths of this had disappeared and there was not enough left to make it worth while to take the picture. His fever had ceased on the third day and he no longer had any difficulty in sleeping. The chancres appeared unchanged. No other treatment had been taken except a potassium chlorate wash for the mouth and a cleansing solution to be frequently injected under the foreskin. Another injection of salicylate of mercury (1 1-5 grains) was administered in the opposite buttock. Nine days later he returned in a most happy frame of mind, as he no longer felt any inconvenience from his disease, the skin was entirely clear, two of the chancres were well and the other two much improved. There had been no return of fever or insomnia. He remarked that the medicine had worked more like magic than he imagined was possible and even now wonders how such a small quantity can be so effective. The injection of 1 1-5 grains was repeated and on his return, a week later the chancres had all healed and the prepuce could easily be retracted. There were a few small erosions in his mouth, from excessive smoking, regardless of my advice to the contrary, and his throat was red and inflamed. Otherwise his condition was most satisfactory. All this improvement had followed three injections of the salicylate of mercury, none being given internally nor did I give iodide of potash or any other preparation. The subsequent history has been uneventful and the disease is kept thoroughly under control by injections about once every seven days.

2. Mr. B. was referred to me on account of the intractible nature of a large indurated chancre involving the greater part of the foreskin and a considerable area on the glans penis around the meatus. Suitable doses of mercury and iodide of potash had been given internally but failed to benefit the condition. Enormous numbers of the spirochaeta pallida were easily demonstrated in the secretion squeezed from the chancre. There was a macular eruption over the body and the lymphatic glands were enlarged. A gonorrhoeal urethritis had also been contracted with the syphilis from the same "Pandora box." The salicylate of mercury was given in 1 grain doses every week, with a dusting powder of equal parts of calomel and stearate of zinc for the chancre and a mouth wash. At the end of two weeks the induration which was so extensive on the penis had disappeared almost entirely and the ulceration had healed. The skin eruption had cleared up a week earlier and the only apparent luetic trouble now was a tonsillo-pharyngitis, which was slower in responding to the treatment and had to be touched a number of times with a 12 per cent chromic acid solution before it was cured. The treatment was continued without the development of any untoward effects or symptoms worth relating.

I have treated a number of other patients by this method with results so prompt and striking that they are always more than willing to submit to the slight aching or pain that follows the injection the first afternoon and night after the treatment. The degree of this pain varies on different occasions, at times being so slight that it is scarcely noticed, but at others is more severe for a short time. The patients find it far less tedious than taking pills or medicine after each meal and the freedom from any indigestion or gastro-intestinal pain or diarrhoea, with the certainty and accuracy of dosage make this treatment much more satisfactory than any other plan so far devised.

METHOD OF TREATMENT. The salicylate of mercury is insoluble and is selected because it is less irritating and more lasting in its action than the other forms of mercury. A convenient method of handling it is in a 10 per cent. suspension in liquid albolene, each drop representing 1-10 grain of the salicylate makes it easy to calculate the exact amount as the doses are changed. Usually 8 minims should be given at the first treatmnet, later it may be increased to 12 or 13, as the condition demands. This can be sterilized by boiling occasionally in a water bath and should always be shaken immediately before using, as it is not in solution. The syringe should be a strong one and of sufficient size to allow the operator to grasp it firmly. The needles must be of large caliber and from 1 1-4 to 1 1-2 inches in length. Each patient should be provided a needle which is to be used on no one else. These may readily be sterilized by boiling or by placing for 8 or 10 minutes in concentrated carbolic acid and then washing in sterile water. The patient reclines face downward on an operating table, with the gluteal muscles relaxed. The buttock is washed with bichloride of mercury and alcohol. Having drawn the proper dose into the syringe and expelled any bubble of air present, the needle is quickly thrust into the soft muscle. Before injecting the medicine, however, the syringe is to be unscrewed from the needle, which is watched for about a minute to see whether or not a blood vessel has been punctured. This is a very rare occurrence, but as any unpleasant results which would follow can be so easily avoided, there is no excuse for not taking this precaution. In case a drop or two of blood flows from the needle it should be partially withdrawn and reinserted. The medicine is injected slowly and the needle quickly removed. The point of the injection is gently massaged for a moment and then the puncture covered with a small piece of cotton and sealed with collodion or adhesive plaster. There should be an intermission in the treatment about every three months and during this period of 3 or 4 weeks especial effort should be made to build up the general health with tonics and iron.

The danger of abscess formation is very slight, as in the 500 injections I have given there has been none. I am firmly of the opinion that the adoption of this plan of treatment in malignanat cases of syphilis would make it unnecessary ever to send such patients to Hot Springs, Ark., and similar resorts. I also believe that in the treatment of the milder forms of the disease it will furnish more satisfactory results than any other method.

1014-15 Century Bldg.

DISCUSSION OF DR. BALLENGER'S REPORT.

Dr. W. B. Armstrong said that he recognized the second patient mentioned by Dr. Ballenger as one he had referred to him and that he could corroborate his statement as to the prompt effect of the intramuscular injections.

---

McGill University was visited on April 16th, with the second serious fire within two weeks. The first fire destroyed the engineering building and the second practically destroyed the medical building, one of the oldest and most valuable on the college grounds. The results of the fire were disastrous, for, in addition to the usual equipment of a college medical building, the museum, one of the best on the continent, was destroyed.

